# Supplementary material for: HIVprotI: an integrated web based platform for prediction and design of HIV proteins inhibitors
Source: J Cheminform. 2018 Mar 9;10:12. doi: 10.1186/s13321-018-0266-y (PMC5845081; doi:10.1186/s13321-018-0266-y)
Supplement: Supplementary file 2 — Additional file 2. Source code of HIVProtI web server. [file 13321_2018_266_MOESM2_ESM.zip › HIVprotI_Source-code/hivprotI/weka-3-6-6/doc/weka/gui/beans/BatchClustererEvent.html]

BatchClustererEvent


---


|  |  |  |
| --- | --- | --- |
| **Overview** \| **Package** \| **Class** \| **Tree** \| **Deprecated** \| **Index** \| **Help** \| **Weka's home** | |  |
| **PREV CLASS**   **NEXT CLASS** | **FRAMES**    **NO FRAMES**     **All Classes** |  |
| SUMMARY: NESTED \| FIELD \| CONSTR \| METHOD | DETAIL: FIELD \| CONSTR \| METHOD |  |


---


## weka.gui.beans Class BatchClustererEvent

```
java.lang.Object
  java.util.EventObject
      weka.gui.beans.BatchClustererEvent
```

**All Implemented Interfaces:**: java.io.Serializable

---

``` public class BatchClustererEvent extends java.util.EventObject ```

Class encapsulating a built clusterer and a batch of instances to
test on.

**Since:**
:   1.0

**Version:**
:   $Revision: 1.4 $

**Author:**
:   Stefan Mutter

**See Also:**: `EventObject`, Serialized Form

---

| **Field Summary** | |
| --- | --- |
| `static int` | `TEST` |
| `static int` | `TRAINING` |


**Constructor Summary**

`BatchClustererEvent(java.lang.Object source,
Clusterer scheme,
DataSetEvent tstI,
int setNum,
int maxSetNum,
int testOrTrain)`
  
          Creates a new `BatchClustererEvent` instance.


| **Method Summary** | |
| --- | --- |
| `Clusterer` | `getClusterer()`             Get the clusterer |
| `int` | `getMaxSetNumber()`             Get the maximum set number (ie the total number of training and testing sets in the series). |
| `int` | `getSetNumber()`             Get the set number (ie which fold this is) |
| `int` | `getTestOrTrain()`             Get whether the set of instances is a test or a training set |
| `DataSetEvent` | `getTestSet()`             Get the training/test set |

**Methods inherited from class java.util.EventObject**

`getSource, toString`

**Methods inherited from class java.lang.Object**

`equals, getClass, hashCode, notify, notifyAll, wait, wait, wait`

**Field Detail**

### TEST

```
public static int TEST
```

---


### TRAINING

```
public static int TRAINING
```


**Constructor Detail**

### BatchClustererEvent

```
public BatchClustererEvent(java.lang.Object source,
                           Clusterer scheme,
                           DataSetEvent tstI,
                           int setNum,
                           int maxSetNum,
                           int testOrTrain)
```

:   Creates a new `BatchClustererEvent` instance.

    **Parameters:**: `source` - the source object: `scheme` - a Clusterer: `tstI` - the training/test instances: `setNum` - the set number of the training/testinstances: `maxSetNum` - the last set number in the series: `testOrTrain` - 0 if the set is a test set, >0 if it is a training set


**Method Detail**

### getClusterer

```
public Clusterer getClusterer()
```

:   Get the clusterer

    :   **Returns:**: the clusterer

---


### getTestSet

```
public DataSetEvent getTestSet()
```

:   Get the training/test set

    :   **Returns:**: the training/testing instances

---


### getSetNumber

```
public int getSetNumber()
```

:   Get the set number (ie which fold this is)

    :   **Returns:**: the set number for the training and testing data sets encapsulated in this event

---


### getMaxSetNumber

```
public int getMaxSetNumber()
```

:   Get the maximum set number (ie the total number of training
    and testing sets in the series).

    :   **Returns:**: the maximum set number

---


### getTestOrTrain

```
public int getTestOrTrain()
```

:   Get whether the set of instances is a test or a training set

    :   **Returns:**: 0 for test set, >0 fro training set


---


|  |  |  |
| --- | --- | --- |
| **Overview** \| **Package** \| **Class** \| **Tree** \| **Deprecated** \| **Index** \| **Help** \| **Weka's home** | |  |
| **PREV CLASS**   **NEXT CLASS** | **FRAMES**    **NO FRAMES**     **All Classes** |  |
| SUMMARY: NESTED \| FIELD \| CONSTR \| METHOD | DETAIL: FIELD \| CONSTR \| METHOD |  |


---
